# Coping Orientation of Academic Community in the Time of COVID-19 Pandemic: A Pilot Multi-Country Survey Study

**DOI:** 10.1177/10541373221088391

**Published:** 2022-04-13

**Authors:** Saeid Zandi, Fereshteh Ahmadi, Önver A. Cetrez, Sharareh Akhavan

**Affiliations:** 1 Department of Social Work and Criminology, Faculty of Health and Occupational Studies, 3485 University of Gävle, Gävle, Sweden; 2 Faculty of Theology, Uppsala University, Uppsala, Sweden

**Keywords:** academic staff, academics, coping strategies, coronavirus epidemic, higher education

## Abstract

In this paper, we have mapped the coping methods used to address the coronavirus pandemic by members of the academic community. We conducted an anonymous survey of a convenient sample of 674 faculty/staff members and students from September to December 2020. A modified version of the RCOPE scale was used for data collection. The results indicate that both religious and existential coping methods were used by respondents. The study also indicates that even though 71% of informants believed in God or another religious figure, 61% reported that they had tried to gain control of the situation directly without the help of God or another religious figure. The ranking of the coping strategies used indicates that the first five methods used by informants were all non-religious coping methods (i.e., secular existential coping methods): regarding life as a part of a greater whole, regarding nature as an important resource, listening to the sound of surrounding nature, being alone and contemplating, and walking/engaging in any activities outdoors giving a spiritual feeling. Our results contribute to the new area of research on academic community's coping with pandemic-related stress and challenges.

## Introduction

Severe acute respiratory syndrome coronavirus-2 (SARS-CoV-2) is rapidly infecting fellow human beings, causing the highly contagious novel coronavirus disease 2019 (COVID-19). The coronavirus epidemic as a worldwide public health crisis has affected our lives in many respects, and its consequences may remain for years to come (Ahmadi et al., 2022c). COVID-19–related lockdown measures, stay-at-home orders, harsh economic realities, and social distancing could have negative effects on mental health (Dubey et al., 2020). A study in the USA demonstrated that personal distancing and stay-at-home regulations during the worldwide crisis were positively correlated with symptoms of psychological disorders (Marroquín et al., 2020).

The COVID-19 outbreak has also severely affected academic community—defined as faculty/staff members and college students– everywhere in the world. Most universities and academic institutions worldwide closed their physical teaching facilities and started distance education/work in response to the outbreak. The pandemic has disrupted university teaching globally, and faculty members and college students have had to get accustomed to new teaching/learning platforms. The COVID-19 epidemic has had negative effects on higher education (Son et al., 2020). In their study, Li et al. (2020) examined the impact of pandemic on psychological health of American and English college students, finding that most of them reported high levels of perceived stress. Son et al. (2020) studied the impact of the coronavirus epidemic on psychological health of university students and demonstrated that 71% of their respondents reported they experienced higher levels of stress and anxiety as a result of the COVID-19 outbreak. In their research, it was also shown that students’ worry about their health, concentration problems, sleep problems, and decreased social interaction are the most important stressors among university students in the era of COVID-19 epidemic.

Recent studies indicate that academics and university students have used various coping strategies to deal with stress, anxiety, and other challenges during the current epidemic (Ahmadi et al., 2022a). For example, Nurunnabi et al. (2020) identified several coping strategies among Chinese college students in the time of coronavirus epidemic in China: seeking social support, avoidance, mental disengagement, and responsiveness to humanitarian issues. Son et al. (2020), investigating coping mechanisms applied by university students in the USA, found that they sought support from others and helped themselves by employing coping strategies. Moreover, Morales-Rodríguez (2021) conducted a study among 180 Spanish university students to investigate the association between fear of coronavirus pandemic and coping strategies. The results of this study revealed that cognitive restructuring, as a coping method, helps the students decrease fear of COVID-19.

As mentioned, coping strategies may help university population better deal with the pandemic-related challenges and stressors. However, little research attention has been paid to meaning-making coping methods in the university community during the COVID-19 pandemic. The main purpose of the study reported here was to investigate the prevalence of the meaning-making coping methods used by the academic community (teachers/researchers, staff and students) to cope with the psychological challenges of COVID-19. The question guiding our study was “Do the meaning-making coping methods used by the university community differ according to the gender, age, work/study status, and place of residence of those using them?.” To that end, the rest of this paper is organized as follows. First, we present the theoretical framework supporting our research. The theoretical framework mainly includes a brief presentation of meaning-making coping, encompassing the whole range of religious, spiritual, and secular existential coping methods. Second, we present our hypothesis. Third, we present our methodology. Fourth, we report the results of our inquiry. Finally, we conclude with a discussion of our results and their implications for further research and practice.

## Theoretical Framework

### Meaning-Making Coping

According to Lazarus and Launier (1978), coping consists of the action-oriented and intrapsychic efforts made by people to manage—minimize, surmount, endure, and reduce—internal and external demands, and associated conflicts, that strain or surpass their resources. Ahmadi and Ahmadi (2018, p. 42) have noted the following:

Coping is regarded (Lazarus & Folkman, 1984, p. 148; Pargament, 1997, p. 89) as a multilayered contextual phenomenon that has several basic qualities. In this regard, Pargament (1997, p. 89) stresses that coping “involves an encounter between an individual and a situation; it is multidimensional; it is multilayered and contextual; it involves possibilities and choices; and it is diverse.” Another dimension of coping is that it constitutes a process that evolves and changes over time.

To help them cope, people use specific resources to orient themselves. Resources such as habits, values, beliefs, personality, and relationships joint constitute orientation systems in terms of which people grasp their worlds (Pargament, 1997). Individuals’ cultures—obviously reflected in these systems—shape how people cope with stress, including that arising from the COVID-19 crisis. This cultural impact has clearly been demonstrated by, for example, Ahmadi et al. (2018), in an examination of coping with crisis.

**Figure 1.**
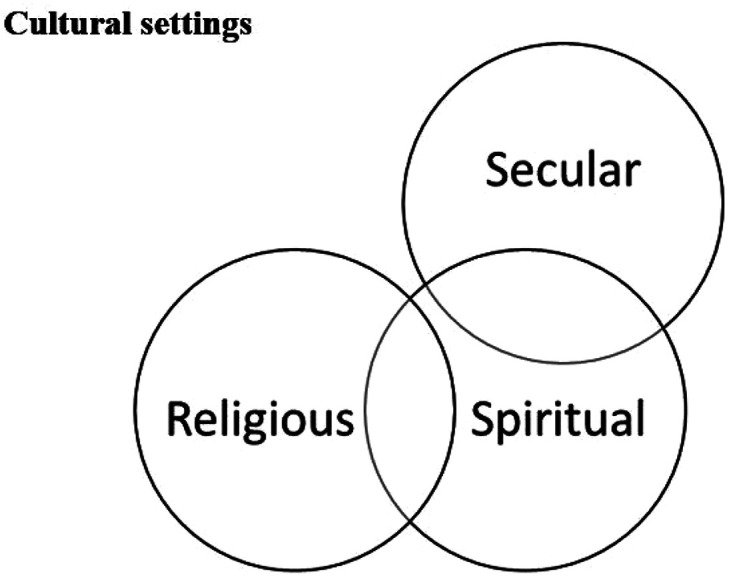
Relation of meaning-making domains (Ahmadi & Ahmadi, 2018, p. 136).

According to Folkman (2008), meaning-focused coping is in its essence, an appraisal-based coping in which the person draws on his or her beliefs (e.g., religious or spiritual), values (e.g., “mattering”), and existential goals (e.g., purpose in life) to motivate and sustain coping and well-being during a difficult time. The existential questions play an important role here. Meaning making process encompasses altering one's own perceptions by reappraising the situation and in this process, some religious/spiritual explanations are found for why stressful life event(s) has occurred or is occurring. “Religious” or “spiritual” coping methods are those that make use of existential awareness. However, many people use non-religious or non-spiritual coping methods, for example, drawing on nature or “internal power”, which are existential and involve meaning-seeking. Such methods were identified by an international research effort in nine cultural settings (Ahmadi, 2006; Ahmadi & Ahmadi, 2018; Ahmadi et al., 2016, 2018; Ahmadi & Zandi, 2021) and by other research as well (la Cour et al., 2012; McDougle et al., 2016). The term “existential coping” is applied to these methods of coping with crisis, because they represent attempts to find internal (e.g., in nature, themselves, or others) rather than transcendent (i.e., in God) resilience. An existential vacuum often arises during crises, when the status quo is challenged and a new order must be born (Ahmadi & Ahmadi, 2018). From this perspective, these spiritual/transcendent and inward/self-directed coping methods are interrelated. Coping via existential meaning-making has little or nothing to do with religious symbology or anything traditionally considered sacred; it can, however, encompass seeking connection with sacred resources in terms of inward sanctification, without referring to God or any traditionally religious features (Ahmadi & Ahmadi, 2021; Ahmadi et al., 2022b). Non-theistic sacred objects are distinct from theistic ones. Unlike the “sacred rings” of Pargament et al. (2017) schema, with the outwardly transcendent as its heart (i.e., theistic sanctification), Ahmadi and Ahmadi (2021) identified another sacred schema in which inward transcendence, via non-theistic sanctification, molds the sacred core. Accordingly, here the full gamut of religious, spiritual, and existential coping methods is captured by the concept of meaning-making coping. Figure 1 shows the relation between meaning-making domains (Ahmadi & Ahmadi, 2018, p. 136). This model differs from those models such as La Cour and Hvidt (2010, p. 1294) in which all these domains (secular, spiritual and religious) are related and have points of connection, i.e., the concepts and topics of each domain overlap to some extent.

It is usually said that people who devote their lives to scientific research may be less religious than the general population (Beit-Hallahmi & Argyle, 1997); therefore, one might expect academics and university students to be less likely to practice their religion than the general population and, therefore, less likely to resort to religious meaning-making coping methods than spiritual and existential coping methods. This leads us to formulate the following hypothesis:

**Hypothesis:** Academic community use secular existential coping methods more often than religious/spiritual ones.

## Methodology

### The Present Study

A quantitative research design was applied for this cross-sectional study. The variables included meaning-making coping methods, gender, age group, work/student status, and place of residence.

### Sample Characteristics

The academic community constitutes a relatively homogeneous population (Fricker, 2008). The academic population in this pilot study consisted of those active in their respective universities, including staff/faculty members and students. For our purpose, we used a convenience sampling method. The inclusion criteria were university staff, students, full or part time, at universities or colleges, aiming at a geographical spread, based on earlier established research networks. We asked our contact persons in different countries, including Austria, Bangladesh, Denmark, Finland, France, Germany, India, Iran, Italy, Malaysia, Malta, Norway, Philippines, Portugal, Saudi Arabia, Singapore, South Korea, Sweden, Switzerland, The Netherlands, Tunisia, Turkey, and UK, to invite the available potential participants to participate in the study and respond to the questionnaire included in the invitation email. A total of 674 individuals responded. The demographic characteristics of our cohort are described in Table 1.

**Table 1.** Demographic Characteristics of Respondents (*n* = 674).

	Variable value	Percent
Gender	Male	34%
	Female	66%
Age group	<35 years old	47%
	35–49 years old	31%
	≥50 years old	22%
Education	University or higher	96%
	High school or similar	4%
Work/student status	Employed	64%
	Student	36%
Civil status	Married	46%
	Divorced	3%
	Engaged	6%
	Single	35%
	Other	9%
Children in family	Children	64%
	No children	36%
Place of residence	Capital	22%
	Medium–large city, not capital	51%
	Small town near a large city	19%
	Small town far from a large city	8%

**Figure 2.**
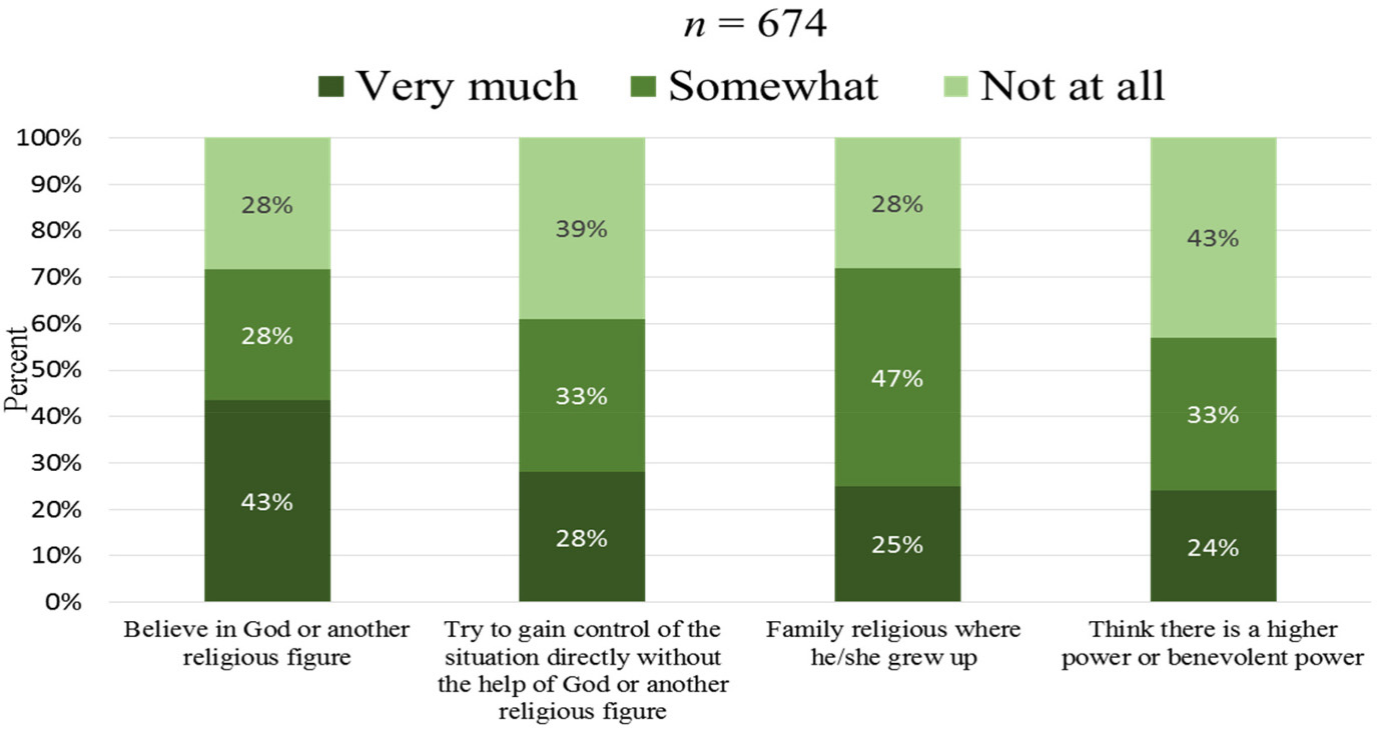
Faith and outlook on life.

Figure 2 shows the informants’ faith and outlook on life. Among the academic community, 71% claimed to believe in God or another religious figure at least somewhat, and about 43% claimed to do so very much; these proportions mirror the shares of those saying that they grew up in religious families. A smaller proportion thought that there was a higher power or benevolent power. In contrast to this, 61% said that they have at least somewhat tried to gain control of the situation directly without the help of God or another religious figure.

### Data

Data were gathered through an open online survey. We used the online survey tool Sunet Survey connected to the University of Gävle. The survey link was e-mailed to faculty members, students, and other staff end of May 2020 and continued till December 2020. In this report, the margin of error is ±4.6 percentage points for results around 50% and 2.8 percentage points for results around 10 or 90%, when the results are based on the whole sample of 674 individuals.

### Measures

The study reported here is based on a modified version of the RCOPE scale covering meaning, control, comfort/spirituality, intimacy/spirituality, and life transformation (Pargament et al., 2000). Five key religious functions constitute the basis of RCOPE. According to Pargament et al. (2000, p. 521), these key functions are: 1. *Meaning*: In the face of suffering and baffling life experiences, religion offers frameworks for understanding and interpretation. 2. *Control*: Confronted with events that push the individual beyond his/her own resources, religion offers many avenues to achieve a sense of mastery and control. 3. *Comfort/Spirituality*: Religion is designed to reduce the individual's apprehension about living in a world in which disaster can strike at any moment. It is difficult, however, to separate comfort-oriented religious-coping strategies from methods that may have a genuine spiritual function. 4. *Intimacy/Spirituality*: Intimacy with others often is encouraged through spiritual methods, such as offers of spiritual help to others and spiritual support from clergy or members. Thus, again, it is difficult to separate out many of the methods that foster intimacy from methods that foster closeness with a higher power. 5. *Life Transformation*: Religion also may assist people in making major life transformations; that is, giving up old objects of value and finding new sources of significance.

The modified RCOPE had a Cronbach's alpha of 0.742 (high) and comprised 15 items rated on four-point Likert scales ranging from 0 (“Never”) to 3 (“Very often”); also, 10 background items were added to the questionnaire (Appendix 1). The scale was validated for content and form in previous research (Ahmadi & Zandi, 2021; Ahmadi et al., 2021).

### Data Analysis

As we used a convenience sampling, there are limits to the generalizability and representativity of the results, why statistical significance was not conducted. We performed various calculations to measure the most frequent coping methods according to age group (i.e., young, middle aged, and older), gender (i.e., female and male), place of residence (i.e., capital city, medium-large city, small town near a large city, and small town far from a large city), and work/student situation (i.e., full-time employment, part-time employment, on-campus student, and distance student). We also performed cluster analysis and factor analysis to answer our research questions. SPSS Statistics 27 was used for data analysis. We should stress that, specifically, we chose to consider participants in total and we did not do much analysis on country difference in this pilot study. We should also mention that the obtained results regard only the participants in this study. We do not have any ambitions to generalize our findings to the whole population.

### Ethical Considerations

We conducted the study according to the Declaration of Helsinki. Our study was approved by the Swedish Ethical Review Authority (Reg. no. 2020/023689). We sent the questionnaire to our contact persons in different universities/colleges. They followed the policies in their respective university/college, sending out the email with the link of the questionnaire to their students and colleagues. In some countries, the approval of the study by the Swedish Ethical Review Authority was sufficient for an internal review at the university, while in others, a committee at the university in question would review and approve the study. In a short letter attached to the survey, the participants were informed about our research, the possibility to withdraw, and the use and preservation of the data. The participants were also informed that responding to the survey would be regarded as giving consent.

## Results

**Figure 3.**
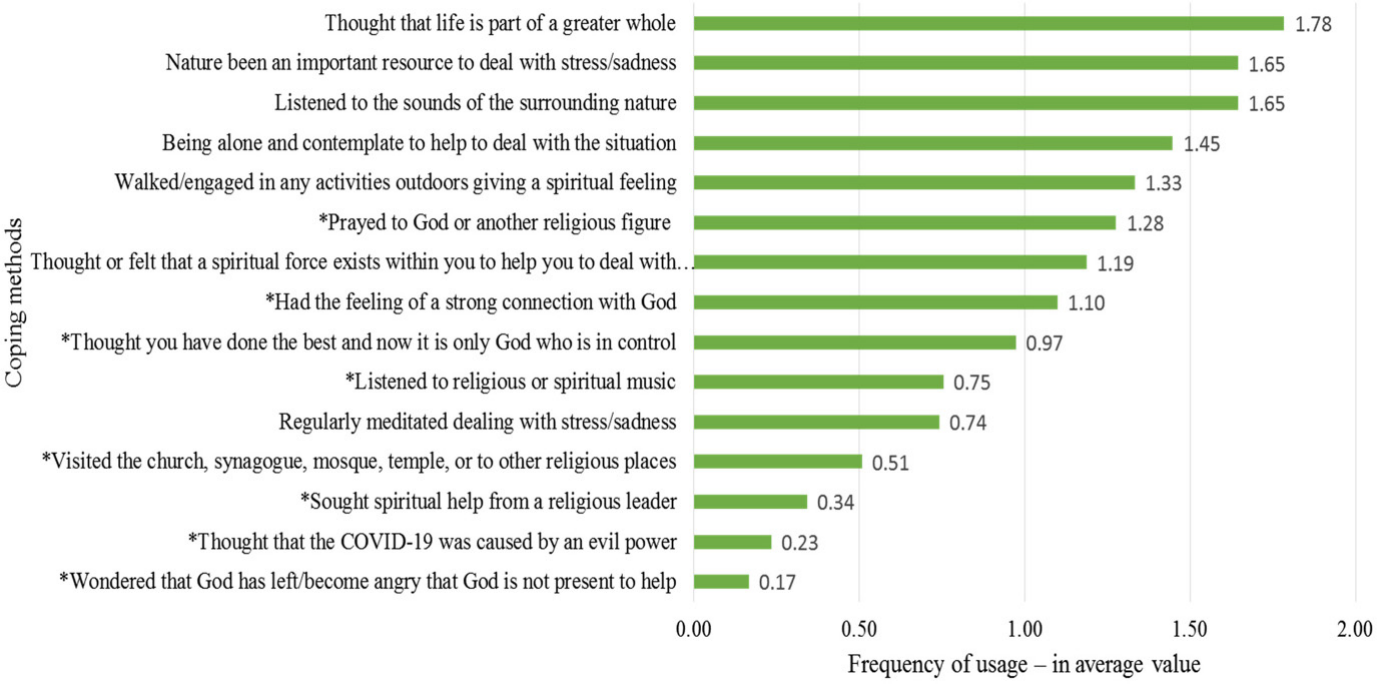
Coping methods used by academic community.

In Figure 3, the coping methods presented here are divided into religious and non-religious coping methods. The religious meaning-making coping strategies have been marked with an asterisk. As this figure indicates, the religious methods were the less popular ones. Of the eight methods less preferred by informants, only one was a non-religious coping method, i.e., meditation, which some might consider a religious coping method. The values of the complete sample in this figure are calculated from how often the respondents used these methods: 0 = never, 1 = sometimes, 2 = quite often, and 3 = very often.

**Figure 4.**
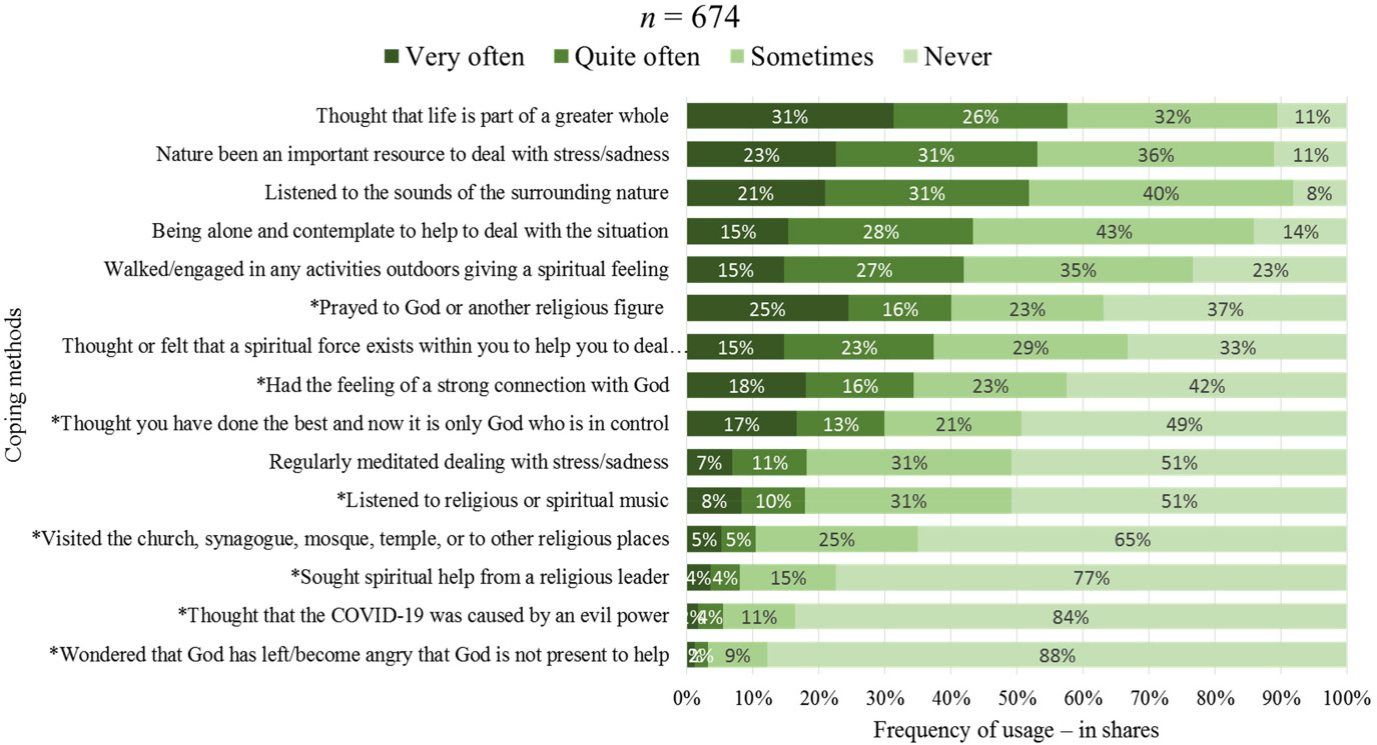
The extent to which the informants have employed various coping strategies.

In Figure 4, we again see a ranking list of the meaning-making coping strategies used, but here considering to what extent the informants have used the different meaning-making coping methods.

### Existential Coping Methods

The coping method *regarding life as part of a greater whole* (1.78) is the leading coping method in this study, used often or very often by up to 57% of respondents. Women and students more than men and university employees had such thoughts.

Academic community turned to nature to cope with the COVID-19 crisis. Over half, 54%, said that nature has often been an important resource when coping with stress and sadness during the ongoing crisis (1.65 index value), and almost as many said that they listened to the sound of surrounding nature to cope with the pandemic situation (1.65 index value). In the ranking list, these two coping methods are the second and third most used, being more used by academic community over 50 years old and those living in small towns far from any large city.

According to our definition presented above, these together with the most common coping method, *regarding life as part of a greater whole* (1.59 index value), are considered secular existential coping methods and are far ahead of other meaning-making coping strategies in terms of how often they were employed.

Another secular existential method for coping with the COVID-19 crisis that was relatively widespread among academic community was to address the dilemma on their own silently. Over 4 in 10 (43%) said that they have often or very often coped with the crisis through being alone and contemplating. This was the fourth most used coping method among academic community. Women, especially those middle aged (35–49 years old), did this relatively often. The next method, *walking/engaging in any activities outdoors giving a spiritual feeling*, is likewise a secular existential coping method. Here also more than 4 in 10 (42%) have often or very often used this coping method.

### Religious Coping Methods

In principle, five of the eight religious coping methods tested here are towards the bottom of the list. The most common religious meaning-making coping strategy was *praying to God or another religious figure*, coming sixth out of fifteen: 41% reported using this religious coping method, but 37% stated that they had never used this coping method.

In general, the results indicate that half of our informants never used any of five of the eight religious coping methods. It was least common to *wonder whether God has abandoned them and to become angry that God is not present*, with under 10% thinking so.

### Factor Analysis of Meaning-Making Coping Methods

To investigate which variables are closely related to one another, we performed a factor analysis. In this study, four factors were generated when this factor analysis was performed (see Table 2). The four factors can be described as *the religious/spiritual dimension*, *religious activity*, *nature/outdoor*, and *negative religious* factors. The religious/spiritual dimension factor has seven variables that are closely associated with each other. The religious activity factor has only three variables, which are often mentioned at the same time as the third factor, the nature/outdoor factor. The fourth factor, the negative religious factor, has only two variables.

**Table 2.** Factor Analysis of Meaning-Making Coping Methods.

	1. Religious/spiritual dimension factor	2. Religious activity factor	3. Nature/outdoor factor	4. Negative religious factor
Have you thought that your life is part of a greater whole?	0.660	−0.066	0.231	0.029
Have you thought or felt that a spiritual force exists in you to help you deal with the situation?	0.736	0.223	0.165	0.129
Has being alone and having the chance to contemplate helped you deal with the situation?	0.571	−0.029	0.345	−0.113
Have you regularly meditated to deal with your stress/sadness or other negative feelings?	0.431	0.182	0.385	−0.033
Have you had the feeling of a strong connection with God?	0.800	0.367	0.055	0.091
Have you prayed to God or another religious figure to make things better?	0.776	0.378	−0.022	0.166
Do/did you think that you have done your best and now it is only God who is in control?	0.756	0.277	−0.060	0.169
Have you sought spiritual help from a religious leader?	0.263	0.746	0.012	0.189
Have you visited the church, synagogue, mosque, temple, or another religious place?	0.085	0.782	0.159	0.052
Have you listened to religious or spiritual music?	0.509	0.625	0.108	0.005
Has nature been an important resource for you in dealing with your stress/sadness or other negative feelings?	−0.040	0.091	0.799	−0.127
Have you listened to the sound of surrounding nature?	0.170	0.016	0.724	−0.015
Have you walked or engaged in any activities outdoors giving you a spiritual feeling?	0.185	0.095	0.685	0.187
Have you thought that the COVID-19 was caused by an evil power?	0.177	0.277	0.080	0.701
Have you wondered whether God has left you or become angry that God is not present to help you?	0.018	−0.022	−0.071	0.862

The factor analysis shows that people have different approaches to religious coping: for certain people, the spiritual aspect of religion, which we call the bright side of religion, is more important in coping (religious/spiritual factor); for others, religious ceremonies and practices are essential in dealing with their stressors (religious activity factor); and for still others, the “dark side of religion” (negative religious factor) (Magyar-Russell & Pargament, 2006) comes into the picture. The nature/outdoor coping methods, which are defined as secular existential coping, do not fall into any of the above religious categories.

### Meaning-Making Coping Methods Used by Coping Method Segments

The frequency of using the different meaning-making coping methods was an important matter addressed in this study. How many used a particular coping method is not in focus here, but rather how often a coping method was used by “natural” groups. To answer this question, we used a cluster analysis, which found three auto-generated segments. This segmentation is based on how often the different meaning-making coping strategies were used and merges participants who answered very similarly regarding how often they employed different meaning-making coping strategies.

**Figure 5.**
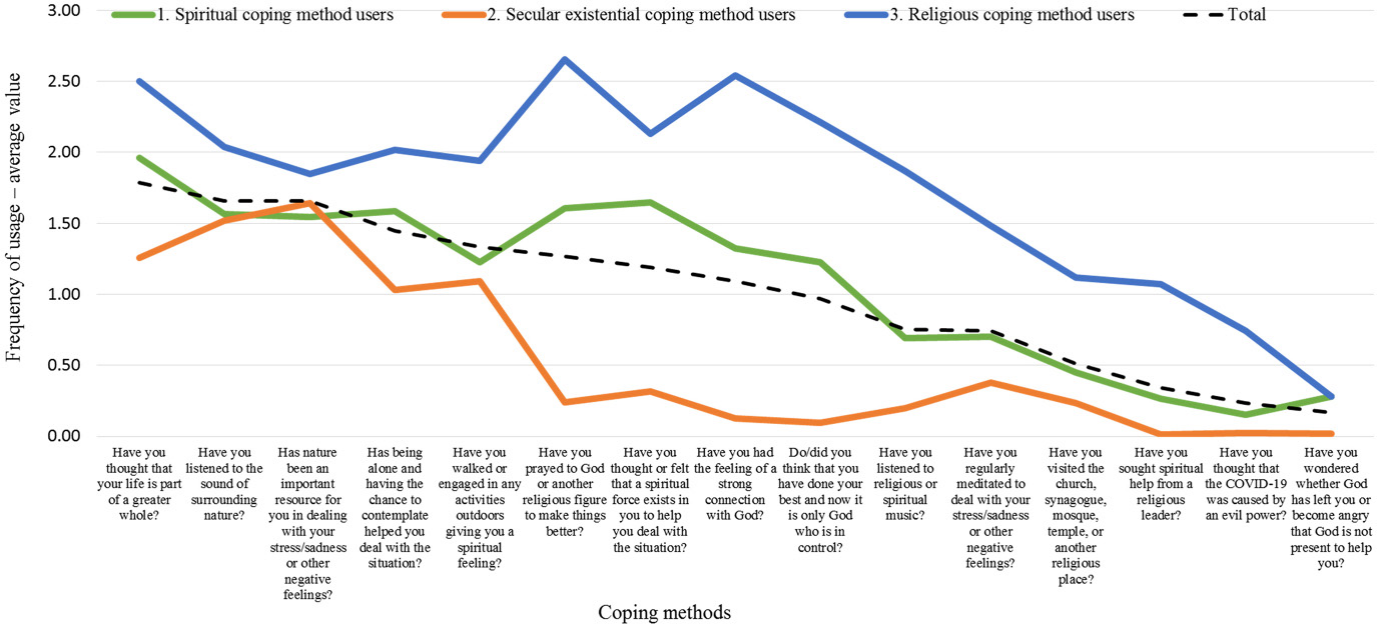
Cluster analysis among academic community.

Figure 5 indicates three segments: *Religious coping method users* with 23% of respondents (the smallest segment); *Spiritual coping method users* with 34% of respondents; and *Secular existential coping method users* with 43% of respondents (the largest segment). Only three segments emerged because the academic community members are too similar within each segment in terms of their frequency of applying different meaning-making coping strategies. It is not ideal to split the academic community into four or more segments, because then the segments would be too similar in terms of the frequency of usage of different coping methods.

The cluster analysis shows that the *Religious coping method users* applied all the meaning-making coping strategies more often than any other segment; notably, members of this group used religious meaning-making coping strategies more often than other coping mechanisms. Two coping methods were used more than others in this group: *praying to God or another religious figure* and *having the feeling of a strong connection with God*.

The second segment comprises the *Spiritual coping method users*. Regarding the frequency of the used coping methods, this segment is the same as of the complete sample. The two most used coping methods in this group were *regarding life as part of a greater whole* and *thinking that a spiritual force exists within them to help them deal with the situation*.

*Secular existential coping method users* applied most of the coping methods but with less than of the complete sample frequency. The most common methods used in this segment were *regarding nature as an important resource to deal with stress and sadness* and *listening to the sound of surrounding nature*.

We found some noteworthy differences between these three groups. *Religious coping method users* were overrepresented among women, accounting for 72% of women versus 66% of the complete sample, and were slightly younger, 53% being under 35 years old versus 47% of the sample. Although no differences were noted in education level, they were more often students (46% vs. the complete sample 36%), lived somewhat more often in a capital city (29% vs. the complete sample 23%), and were overrepresented among those from Iran (51% vs. the complete sample 29%). Moreover, 91% believed in God very much, compared with the complete sample of 43% in this study, and much more often came from religious families. Their self-rated health was more often excellent (25% vs. the complete sample 18%). Their marital status followed the complete sample values.

*Spiritual coping method users*—a segment of the complete sample in terms of both size and how often different coping methods are used—has similar shares of men and women. They were somewhat younger than the complete sample, 53% being under 35 years old compared with the complete sample of 47%. Their education level was like the complete sample, but they were more often students (43% vs. the complete sample 36%). They were like the complete sample regarding the place of residence, but much more often lived in Iran (46% vs. the complete sample 29%) and more seldom lived in Sweden (26% vs. the complete sample 43%). A slightly larger share was religious (59% vs. the complete sample 43%); they were like the complete sample in health status and similar to the complete sample in marital status.

The biggest segment, *Secular existential coping method users*, follows the complete sample for gender in the study, comprising 65% women. Older people, 50 years of age and above, were slightly overrepresented, comprising 30% of the segment versus 22% of the whole sample. The share of those with a university education was almost the same as the complete sample at 95%. The share of employed in this segment was larger than the complete sample for the sample, i.e., 74% versus 64%. They followed the complete sample for the kind of city they lived in. Concerning the country of residence, those living in Sweden were overrepresented, constituting 72% of this segment versus 43% of the sample, and those living in Iran were underrepresented, i.e., 3% versus 28%. Sixty-three percent of this segment's members did not believe in God (vs. 29% of the sample) and they came much more rarely from religious families. They were like the complete sample in health and in marital status.

## Discussion

This study intended to explore and identify the meaning-making coping methods used by the university community during the COVID-19 epidemic. The results indicate that although 71% of informants believed in God or another religious figure at least somewhat, 61% reported at least somewhat trying to gain control of the situation directly without the help of God or another religious figure. In general, the results indicate that half of the respondents never used any of five of the eight religious coping methods. Concerning religious coping, the factor analysis revealed different approaches: the first approach emphasizes the spiritual aspect of religion (the bright side of religion) in coping (religious/spiritual factor); the second approach emphasizes religious ceremonies and practices as essential in coping (religious activity factor); and the third approach emphasizes the “dark side of religion” (negative religious factor) (Magyar-Russell & Pargament, 2006). The ranking of the used methods indicates that the first five coping methods belong to the group of non-religious coping methods, defined as secular existential meaning-making coping (Ahmadi et al., 2018): *regarding life as a part of a greater whole*, *regarding nature as an important resource*, *listening to the sound of surrounding nature*, *being alone and contemplating*, and *walking/engaging in any activities outdoors giving a spiritual feeling.* The study apparently supports our hypothesis that secular existential coping methods are used more often than religious ones among academic community.

As our target population was from academic communities, they were expected to be more educated and intellectual than the general population. Also, considering that it is often said that people who devote their lives to scientific research are less religious as compared to the general population (Beit-Hallahmi & Argyle, 1997), one might expect academic community to be less likely to practice their religion than the general population and less likely to resort to religious meaning-making coping methods than spiritual and existential coping methods. However, there are inconsistent findings regarding the correlation between level of education and religiosity. Several studies provide evidence that educated people are less likely to have faith in God (e.g., Arias-Vazquez, 2012; Hungerman, 2014; Johnson, 1997; Pew Research Center, 2018; Sherkat & Ellison, 1999; Zuckerman et al., 2013), whereas other studies have found that education appears to increase religious practice (e.g., Glaeser & Sacerdote, 2008; Iannaccone, 1998). The World Values Survey (Wave 7, 2017–2020; see Haerpfer et al., 2020) captures a more nuanced picture of the relationship between educational level and religiosity, measured by its importance in life; in general, slightly over half of those with the highest education state that religion is rather or very important, while even more of those with lower education levels, over two thirds, state that religion is rather or very important. Ganzach et al. (2013) attempted to resolve these findings by proposing that education positively affects religiosity if the religious background is strong, but negatively affects it if the religious background is not strong. Altogether, as indicated, more of the literature demonstrates that higher levels of education and low religious orientations overlap, but that there are still highly educated strong believers.

The scholarly results present a scattered picture of the relationship between educational level and degree of religiosity. For our purposes, let us look more closely at Sweden, Iran, and Turkey, based on data from the World Values Survey (Wave 7, 2017–2020; see Haerpfer et al., 2020). In Sweden, those with lower and higher education differ little regarding religion, approximately one third of both groups stating that it is rather or very important in life. In Iran, almost all respondents state that religion is rather or very important, but slightly more of those with lower education do so. In Turkey also a clear majority say that religion is rather or very important, but again, slightly more of those with lower education do so. A main problem when measuring religiosity is that different dimensions of religiosity can be measured, be it faith in God, religious practice, or importance in life. Furthermore, religion is understood differently in different cultures. An understanding of religiosity as having a personal inner faith, or view of life, has different connotations in terms of importance in life, compared with an understanding of religion as a system that regulates everyday life practices or interactions with other people. Again, to cite an example from the World Values Survey, when religion is considered a meaning-making system, the outcome is different. In Iran, a clear majority, three quarters, of those with higher education understand the meaning of religion as doing good to other people, while a small minority say that the meaning of religion is to follow norms and ceremonies (World Values Survey, Wave 7; see Haerpfer et al., 2020). In Turkey, the situation is different, and approximately half of those with higher education think that the meaning of religion is to do good to other people and half think that it is to follow norms and ceremonies. This interesting divergence between two Islamic countries merits analysis from a cultural perspective. Data from Sweden are not available for the same time period, but earlier data from 2010–2014 (World Values Survey, Wave 6; Inglehart et al., 2014) reveal that a clear majority, almost nine in ten, of those with higher education think that the meaning of religion is to do good to other people. In this respect, the data from both Sweden and Iran are linked more closely to the spiritual aspect of religion in coping (i.e., the religious/spiritual factor) in the factor analysis.

We should mention that similar earlier studies of meaning-making coping among cancer patients and bereaved parents (Ahmadi & Ahmadi, 2015, 2017; Ahmadi et al., 2018, 2020, 2021; Ahmadi & Zandi, 2021; Cetrez et al., 2020) confirm our results. These studies show that the application of religious coping methods is not highly prevalent among people with higher education, who tend to use more existential coping methods.

Beit-Hallahmi (2015) showed that academic community might be less religious because of “a process of selection and self-selection, a process starting in childhood and channeling persons who are highly intelligent, critical, independent, and confident towards the academic world” (p. 104). In other words, it is not the academic degree or level that creates its own secularity, but rather that young secular persons are more likely to choose an academic life (Beit-Hallahmi, 2015). It seems that demographic factors and the religiosity of the family in which one is raised may affect the selection of coping methods in difficult times of life.

Another aim of this study was to map the differences in meaning-making coping strategies employed by academic community in terms of the variables age group, gender, work/student status, and place of residence. Comparing these four variables shows that the coping method *regarding life as part of a greater whole*, which was used by most informants, was also usually the coping method most commonly used by different subgroups—i.e., by men, women, the two younger age groups, those with university education, those employed or students, and those living in a capital city. The most common religious meaning-making coping strategy, *praying to God or another religious figure*, was most common among students, those under 35 years old, and women.

Another finding of our study is that women were somewhat overrepresented among religious coping method users, who were also slightly younger and more often students living in capital cities. According to previous research, demographic factors, marital status, age, and presence of children in the family are the strongest predictors of religious differences among scientists. Specifically, religiosity in one's childhood home was the most important factor that predicted present religiosity among a sample of scientists (Ecklund & Scheitle, 2007).

## Conclusions

This study revealed that secular-existential coping appears to be most favoured by a—globally constituted—academic community. Also, it was demonstrated that religious coping methods are predominantly engaged in by women (echoing and confirming extant research on women's higher levels of religiosity than men's). In the studied population, highly academic and adhering strongly to existential coping methods, an inward sense of sacred transcendence guided their coping methods. Ahmadi and Ahmadi (2021) maintained that there is hardly any point of connection between secular existential meaning-making coping and traditional sacred contexts. Secular meaning-making coping can thus overlap with a search for connectedness with a sacred source in terms of inward sanctification without relating to God or any traditional religious context. It is in this regard that these scholars distinguish between theistic and non-theistic sacred objects and present a model of “sacred rings” that is in contrast to the set of sacred rings presented by Pargament et al. (2017). Pargament's model of sacred rings has the outwardly transcendent as its sacred core (i.e., theistic sanctification), while in Ahmadi and Ahmadi's model, inward transcendence shapes the sacred core (i.e., non-theistic sanctification). This latter model may help us better understand why academic community members, even those who are believers, are often inclined to apply secular–existential coping methods rather than religious ones. Indeed, the problem is not denying the sacred or the importance of sanctifying objects and issues in coping, but rather how to view the sacred. A coping method such as visiting a church or reading the Bible with its outwardly transcendent connotations is more difficult to internalize for an academic than is a coping method such as immersing oneself in nature with its inwardly transcendent connotations. The need for scientific verification has always been important for scientists and academics in order to accept and internalize religious beliefs.

Our quantitative survey-based study has certain limitations in terms of the very limited size of the sample, research design, and sampling strategy. Moreover, the sample skewed female. Generalization of the present findings to different populations and settings should not be done. As we could not conduct further quantitative analyses of gender, age, living area, or educational level within each country (using ANOVA), we chose to treat the responses as representing our academic sample as a whole, without making any country-specific specifications. Despite these limitations, we believe that our results may contribute to the new and important area of COVID-19 and coping methods among university community, not the least as the data were gathered during the worldwide pandemic situation, rather than as part of a retrospective study.

Self-care can be a buffer against the mental and physical health impacts of COVID-19 pandemic- related chronic stress (Luis et al., 2021). As Vazquez Morgan (2021) argues, a need exists for academic institutions to evaluate challenges to self-care and wellbeing in college students, and to develop (campus-based) interventions aimed at improvement of self-care in them. It is therefore recommended that future studies investigate the place and influence of coping orientation on self-care among those within the global academic community.

Preferred meaning-making coping strategies differ by age group, gender, work situation, and area of residence, and this merits further investigation. In addition, extending knowledge regarding the importance of different meaning-focused coping methods among university community may help the university counsellors and psychologists to more efficiently assist college students and academics during times of tragedy and crisis.

## Supplemental Material

sj-docx-1-icl-10.1177_10541373221088391 - Supplemental material for Coping Orientation of Academic Community in the Time of COVID-19 Pandemic: A Pilot Multi-Country Survey Study Click here for additional data file. Supplemental material, sj-docx-1-icl-10.1177_10541373221088391 for Coping Orientation of Academic Community in the Time of COVID-19 Pandemic: A Pilot Multi-Country Survey Study by Saeid Zandi, Fereshteh Ahmadi, Önver A. Cetrez and Sharareh Akhavan in Illness, Crisis & Loss
