# Supplementary material for: Coping Orientation of Academic Community in the Time of COVID-19 Pandemic: A Pilot Multi-Country Survey Study
Source: Illn Crises Loss. 2022 Apr 13;31(3):504–24. doi: 10.1177/10541373221088391 (PMC9014345; doi:10.1177/10541373221088391)
Supplement: sj-docx-1-icl-10.1177_10541373221088391 - Supplemental material for Coping Orientation of Academic Community in the Time of COVID-19 Pandemic: A Pilot Multi-Country Survey Study [file sj-docx-1-icl-10.1177_10541373221088391.docx]

**Coping with COVID-19**

# **Consent**

1. I have been informed about the study “Coping with COVID-19” and I give consent to participate in this study:

Yes 0 No 1

2. I give consent that my answers will be handled as described in the information letter:

Yes 0 No 1

# **Coping**

**What do/did you do to handle the situation during the “COVID-19 time”, if you feel/felt stressed, sad, or depressed or have/had other reactions?**

|  | Never – 0 | Sometimes –1 | Quite often –2 | Very often –3 |
| --- | --- | --- | --- | --- |
| 3. Have you thought that your life is part of a greater whole? | 0 | 1 | 2 | 3 |
| 4. Have you thought or felt that a spiritual force exists in you to help you deal with the situation? | 0 | 1 | 2 | 3 |
| 5. Has nature been an important resource for you in how to deal with your stress/sadness or other negative feelings? | 0 | 1 | 2 | 3 |
| 6. Has being alone and having the chance to contemplate helped you deal with the situation? | 0 | 1 | 2 | 3 |
| 7. Have you listened to the sounds of surrounding nature? | 0 | 1 | 2 | 3 |
| 8. Have you walked or engaged in any activities outdoors that give/gave you a spiritual feeling? | 0 | 1 | 2 | 3 |
| 9. Have you regularly meditated to deal with your stress/sadness or other negative feelings? | 0 | 1 | 2 | 3 |
| 10. Have you sought spiritual help from a religious leader? | 0 | 1 | 2 | 3 |
| 11. Have you thought that COVID-19 was caused by an evil power? | 0 | 1 | 2 | 3 |
| 12. Have you wondered whether God has left you or become angry that God is not present to help you? | 0 | 1 | 2 | 3 |
| 13. Have you had the feeling of a strong connection with God? | 0 | 1 | 2 | 3 |
| 14. Have you visited a church, synagogue, mosque, temple, or other religious place? | 0 | 1 | 2 | 3 |
| 15. Have you prayed to God or another religious figure to make things better? | 0 | 1 | 2 | 3 |
| 16. Have you listened to religious or spiritual music? | 0 | 1 | 2 | 3 |
| 17. Do/did you think that you have/had done your best and now it is only God who is in control? | 0 | 1 | 2 | 3 |
| 18. Have you tried to gain control of the situation directly without the help of God or another religious figure? | 0 | 1 | 2 | 3 |
| 19. Do you believe in God or another religious figure? | 0 | 1 | 2 | 3 |
| 20. Do you think there is a higher power or benevolent power? | 0 | 1 | 2 | 3 |
| 21. Would you say that you come from a religious family? | 0 | 1 | 2 | 3 |

**Background questions**

We end by gathering some background information, which is needed for our analysis.

28. What is your current work/student status?

1 Employed full-time

2 Employed part-time

3 On-campus student

4 Distance-learning student

29. What year were you born? …….

30. What is your gender?

1 Male

2 Female

3 Neither of the above

31. What is your highest education level?

1 Lower than elementary school

2 Elementary School or equivalent

3 High School or equivalent

4 University or equivalent

32. What is your current civil status?

1 Married

2 Divorced

3 Engaged

4 Widowed

5 Single

6 other (please briefly specify)………………

33. Do you have children?

 0 Yes

 1 No

34. What characterizes the place you live?

 0 Capital

 1 Medium–large city, not capital

 2 Small town close to a large city

 3 Small town far from a large city

35. What is your country of birth? (please write).................................

36. Country of residence: (please write) ……………………………..
